# Evaluation of the Correlation between CD44, Tumor Prognosis and the 5-Year Survival Rate in Patients with Oral Tongue SCC

**Published:** 2016-11

**Authors:** Reza Kaboodkhani, Ebrahim Karimi, Mohammad Taghi Khorsandi Ashtiani, Safoura Kowkabi, Mohammad Reza Firouzifar, Farzad Yazdani, Nasrin Yazdani

**Affiliations:** 1 *Department of Otorhinolaryngology and Head and Neck Surgery, Shiraz University of Medical Sciences, Shiraz, Iran.*; 2 *Otorhinolaryngology Research Center, Tehran University of Medical Siences, Tehran, Iran.*; 3 *Neurologist, Shiraz University of Medical Sciences, Shiraz, Iran.*; 4 *Department of Otorhinolaryngology, Amiralam Hospital, Tehran University of Medical Siences, Tehran, Iran.*; 5 *Department of Pathology, Amiralam Hospital, Tehran University of Medical Siences, Tehran, Iran.*

**Keywords:** CD44, Tongue SCC, Survival

## Abstract

**Introduction:**

90% of the tumors in the head and neck are squamous-cell carcinomas (HNSCC), which have overall 5- year survival rate between 50% -60%. CD44 has been shown to be associated with the prognosis.

**Materials and Methods:**

Biopsy specimens of 51 patients with oral tongue SCC were evaluated by Immunohistochemistry (IHC) for the CD44 antibody.

**Results:**

There was no significant correlation between CD44 and survival (P=0.77), age (P=0.4), CD44 and lymph node metastasis (P=0.87), sex (P=0.947), smoking (P=0.287) and tumor size (P=0.813). However, there was significant correlation between smoking and survival.

**Conclusion:**

There are widespread discrepancies among the findings in the literature regarding the prognosis of CD44 expression in OCSCC. Our study shows that the expression of CD44 is not a marker of aggressive behavior in oral tongue SCC. Consequently, CD44 cannot be considered as handy tool to establish the tumor behavior, prognosis and 5- year survival rate of these tumors.

## Introduction

Head and neck squamous cell carcinomas (HNSCC) are the sixth most common cancers worldwide with an estimated annual burden of 3,55,000 deaths and 6,33,000 incident cases (1). The incidence of the majority of HNSCC subsets (i.e., cancers of lip, oral cavity, larynx, hypopharynx, and nasopharynx) has decreased significantly during the past 20 years in the United States and other developed countries, mainly because the decline in cigarette smoking (2,3). The overall 5- year survival rate of HNSCC patients is reported to be between 50% - 60%. Over the last few years, the prognosis of many types of cancer has significantly improved because of advances in diagnosis and treatment. Despite these advances, the prognosis of advanced HNSCC has not changed as much.

The TNM stage of tumors can help to determine the prognosis of the patient and is the main factor for planning treatment. The fact that some patients with same staging have different prognosis suggests that factors like biologic behavior may play role in the outcome. To date, no generally accepted biomarker is found to show more invasive course in HNSCC. Therefore, we need to know more about the cellular mechanisms involved in HNSCC cell proliferation to better highlight the aggressive types and choose proper treatment regimen (4).

Overexpression of CD44 in tumor cells has been associated with increased radioresistance and local recurrence rate (5-7) While in some other studies , downregulation of CD44 , was a marker of unfavorable prognosis, especially in oral cavity squamous-cell carcinomas (8, 9). These contrasting results shows us that there are mechanisms in CD44 mediated pathways yet not understood (10).The aim of this study is to determine if there is any correlation between CD44expression and 5- year survival rates of patients. 

## Materials and Methods

Pathology specimens of 51 patients with oral tongue SCC were evaluated. All patients were surgically treated for their primary tumor and neck lymph nodes were treated accordingly. Tissue blocks were made as standard guidelines suggest.(11) Sections were incubated with the primary anti-CD44 (Lyophilized Novacastra Variant 3 Company). Staining was completed according to manufacturer. Positive and negative tissue control were also done for each process.

Staining results were evaluated by a single qualified pathologist who was blinded to sample characteristics. The immunostaining pattern of CD44 was classified as follows: group1: positive staining for CD44. Group 2: negative staining for CD44. The correlation between CD44 immuno- expression with clinicopathological findings (TNM stage, positive lymph node metastases, smoking, 5-year survival, age, sex) was analyzed by Pearson’s Chi-square test(or Fisher’s exact test) and the independent t-test according to qualitative and quantitative data respectively. We lost 4 patients to follow up. 

## Results

This study evaluated the association between CD44 and the 5 - year disease- free survival rate in 51patients with oral tongue SCC. Of these, 24 patients (47%) were male and 27 patients (53%) were female. The mean age was 57.8 (23 min - 84 max), none of whom had previously received treatment. 25.5% of patients were smokers.

Based on the T stage, 9 (17.6%) patients were T1, 19(37.3%) T2, 17(33.3%) T3 and 6 (11.8%) T4. 18(35.3%) patients had clinical lymphadeno- pathy at the time of diagnosis. 25 (49%) patients had lymph node metastasis according to the pathology reports. The data of 4 patients (8%) were unavailable. The Disease -specific survival rate was 43%. There was no significant correlation between CD44 and survival (P=0.77), age (P=0.4), clinical LAP (P = 0.155), lymph node metastasis (P=0.87), sex (P=0.947), smoking (P= 0.287), and tumor size (P=0.813). Survival of the patients in relation with Lymphadenopathy, tumor stage, smoking habits and neck metastasis is summarized in table 1. All of the above mentioned variants had significant correlation with survival. 

**Table 1:** Survival in relation to lymphadenopathy, smoking, tumor size, and neck metastasis

		LAP		Sig	Smoking		Sig	Tumor size				Sig	Neck metastasis		Sig
		+	-	0.001>*	+	-	0.011*	T1	T2	T3	T4	0.01*	+	-	0.001>*
Survival	Unknown		4			4		1	3				1	3	
	Alive	2	20		2	20		7	10	4	1		2	20	
	Dead	16	9		11	14		1	6	13	5		22	3	

* P<0.05 was considered statistically significant

There was no significant correlation between age and pathological lymph node metastasis (P=0.07) and clinical lymph node metastasis (P=0.3). The mean age of patients with a greater tumor size (3,4) is more than that of patients with a smaller size. There was a significant direct correlation between age and tumor size (P=0.04).

There was a significant correlation between clinical LAP and lymph node metastasis (Table.2). There was no significant correlation between clinical LAP and sex or smoking.

**Table 2:** Correlation of clinical LAP with lymph node metastasis

	Neck		Total
	+	-	
Lap + -	17	1	18
	8	25	33
Total	25	26	51

* LAP Neck Crosstabulation

There was a significant correlation between clinical LAP and tumor size (Table.3), between smoking and lymph node metastasis (Table.4), and between tumor size and lymph node metastasis (Table.5).

**Table 3:** Correlation of clinical LAP with tumor size

		Neck		Total
		+	-	
T	T T2 T3 T4		9	9
		3	16	19
		10	7	17
		5	1	6
Total		18	33	51

* T LAP Crosstabulation

**Table 4:** Correlation of smoking with lymph node metastasis

		Neck		Total
		+	-	
Risk Total	+ -	11	2	13
		14	24	38
		25	26	51

* Risk Neck Crosstabulation

**Table 5:** Correlation of tumor size with lymph node metastasis

		Neck		Total
		+	-	
T	T1 T2 T3 T4		9	9
		5	14	19
		15	2	17
		5	1	6
Total		25	26	51

* T Neck Crosstabulation

## Discussion

HNSCC is one of the most common cancers. Despite recent advances in diagnosis and treatment, the prognosis has not changed significantly (12-15). 

The 5-year survival rate of patients with neck lymph node involvement still remains at 53%. Cervical lymph node and distant metastasis are the major causes of treatment failure in these patients (16-20).

 To date no reliable cell marker is found to help differentiate those HNSCC with poorer and better outcomes (10). 

We compared the relative CD44 mRNA expression of patients with age, sex, smoking, tumor size, clinical and pathological LAP and 5 years survival rate. In our study the relative CD44 mRNA expression of patients did not correlate with age, sex, history of smoking, tumor size, clinical and pathological LAP and 5 years survival rate.

There was significant indirect correlation between survival, age, tumor size, clinical LAP and pathological lymph node metastasis. Survival was lower with older age, greater tumor size and positive LAP and lymph node metastasis. Also there was significant association between survival, sex and smoking. Males and smokers had less survival than females and non-smokers. Smokers had greater tumor size and more LAP. Larger tumors had statistically more metastasis than smaller ones. There was not significant correlation between age and lymph node metastasis but there was significant direct correlation between age and tumor size, patients with older age refer with larger tumor size that is might be due to delayed referral to physician. Pathological lymph node metastasis was reported in most patients with cervical LAP in physical examination that was statistically meaningful. 

Kokko et al (10) evaluated associations between tumor CD44 expression and smoking, heavy alcohol consumption, histological grade, and TNM staging. When all HNSCC subsites were studied, they found significant correlation between CD44 overexpression and decreased 5- year survival rates (P<0.001). Moreover, in patients of with SCC of the oro-and hypopharynx and larynx, a significant correlation was seen between intense CD44 expression and poor 5- year survival rates (P< 0.001). However, in patients suffering from oral cavity SCC there was no significant correlation. the Kokko study reported a significant association between tumor CD44 overexpression and heavy smoking of more than 10- pack years (P=0.009). It was concluded that, in addition to staging, CD44 overexpression could be considered as an indicator of aggressiveness and a prognostic factor in pharyngeal and laryngeal SCC. Therefore, it could assist in treatment selection.

Chou et al (21) reported that patients with CD44 rs187115 variant genotypes (AG+GG) had higher risk of oral cancer development. In comparison to WT carriers, they found a greater chemoresistance to advanced- to late-stage oral cancer. Chou reported that gene–environment interactions between CD44 polymorphisms and betel quid chewing and tobacco smoking increased susceptibility to developing oral cancer. It was concluded that CD44 rs187115 polymorphism may be considered as a predicting factor of the clinical stage in OCSCC patients. Stoll et al (8) suggested a 39.4% decrease in expression of CD44 in their cases of oral and oropharyngeal SCC. They could not show a direct correlation between cervical lymph node involvement and CD44 expression. However, the overall -survival and disease-free-survival of their patients were worse than those with positive CD44 expression.

Mostaan et al (11) reported a significant correlation between reduced expression of CD44 and cervical lymph node metastasis, but no correlation with tumor differentiation.

Kusunen et al (22) found a correlation between lesser expression of CD44 and worse tumor staging and unfavorable outcomes. Okamoto (23) suggested that CD44 can bind to hyaluronan and probably stabilize its connection with the extracellular matrix. So lesser level of CD44 expression may increase the metastasis rate and shorten the survival.

Finally, Lin et al (24) reported a significant association between high pretreatment CD44mRNA levels and a poorer prognosis (P<0.001). 

These controversial results may be because of heterogeneity in tumor cells, which may be because of tumor microenvironment (8,23). However, when immunoreactivity is specifically evaluated in the invasive tumor front (ITF), it may statistically show a significant relationship with the invasive front grading score, primary tumor pathology, tumor thickness, and poor survival (23). 

## Conclusion

There are widespread conflicting findings in the literature regarding the prognosis of CD44 expression in relation to OCSCC. Our study shows that that the expression of CD44 is not a marker of aggressive behavior in oral tongue SCC. Consequently, CD44 cannot be considered as handy tool to establish the tumor behavior, prognosis and 5- year survival rate of these tumors.
